# Resting energy expenditure changes related to different sedation levels in critically ill patients undergoing invasive mechanical ventilation

**DOI:** 10.3389/fmed.2026.1699235

**Published:** 2026-04-30

**Authors:** Chengqing Mei, Zhenglong Ye, Hui Zou, Zhiqing Hu, Yong Wu, Shangxiang Liu

**Affiliations:** Department of Critical Care Medicine, Nanjing Jiangbei Hospital, Nanjing, Jiangsu, China

**Keywords:** critical illness, degree of sedation, indirect energy metabolism, mechanical ventilation, resting energy expenditure

## Abstract

**Objective:**

The study aimed to assess the effects of resting energy expenditure (REE) on critically ill individuals requiring mechanical ventilation with different degrees of sedation.

**Design:**

A prospective self-controlled study was conducted involving 74 critically ill patients with clear consciousness who required mechanical ventilation from September 2023 to September 2024. Sedation was induced and maintained using midazolam, and various levels of sedation were achieved by adjusting the administered dose of midazolam. The level of sedation was evaluated using the Richmond Agitation-Sedation Scale (RASS): mild sedation (RASS score 0–1), moderate sedation (RASS score −2 ~ −3), and severe sedation (RASS score −4 ~ −5). Indirect calorimetry (IC) was employed to assess REE across varying levels of sedation.

**Results:**

With the increase in sedation, oxygen consumption, carbon dioxide production, and REE gradually decreased. There were significant variations in REE among severe sedation (1,140 ± 108 kcal/d), moderate sedation (1,236 ± 137 kcal/d), and mild sedation (1,347 ± 129 kcal/d) (*p* < 0.05). There were no notable differences in the respiratory quotient (RQ) across different degrees of sedation (*p* > 0.05). Multiple logistic regression analysis revealed that sedation, sex, age, and body mass index (BMI) were independent factors influencing REE.

**Conclusion:**

Sedation affected energy expenditure in critically ill individuals who underwent mechanical ventilation, and REE decreased gradually with increasing depth of sedation.

## Introduction

Critically ill patients require a sufficient amount of nutritional therapy to meet their metabolic and nutritional needs throughout their illness. It is of the utmost significance to ascertain energy requirements (1). Resting energy expenditure (REE) refers to the energy expended by the body at rest. In patients with critical illness, the reduced level of physical activity results in REE being nearly equivalent to total energy expenditure (TEE) (2). Indirect calorimetry (IC) quantifies the body’s oxygen consumption (VO_2_) and carbon dioxide production (VCO_2_), subsequently determining REE through the application of the adjusted weir equation. This approach is the most effective method for assessing and managing energy expenditure while optimizing nutritional strategies (3). Under the guidance of IC, implementing targeted nutritional interventions for patients can prevent energy deficiency or overfeeding and enhance patient outcomes (4). Personalized sedation therapy is a crucial component of the comprehensive management of patients with critical conditions, particularly those on mechanical ventilation (5, 6). However, the impact of sedation therapy on REE in this population remains unclear. This research seeks to explore the impact of different sedation doses, assessed using IC, on REE in mechanically ventilated critically ill patients.

## Materials and methods

### Participants

Between September 2023 and September 2024, patients were carefully selected from the intensive care unit (ICU) of Nanjing Jiangbei People’s Hospital, specifically those in severe critical condition requiring mechanical ventilation while maintaining consciousness, to serve as participants of this study. For patients receiving parenteral or enteral nutrition, the infusion rate was maintained consistently both before and during the measuring period.

#### Inclusion and exclusion criteria

The inclusion criteria were as follows: ① age > 18 years; ② critically ill patients requiring invasive mechanical ventilation; and ③ presence of at least one organ dysfunction, characterized by a sequential organ failure assessment (SOFA) score of ≥ 2 points. The exclusion criteria were as follows: ① pregnant or postpartum patients; ② brain death or impaired consciousness; ③ patients undergoing blood purification (a medical procedure that uses specialized equipment to remove harmful substances from the bloodstream) or presence of pneumothorax; ④ patients with positive end-expiratory pressure (PEEP) > 12 cm H_2_O or fraction of inspired oxygen (FiO_2_) > 0.6; ⑤ neurological or muscular disorders, including myasthenia gravis and Guillain–Barré syndrome; and ⑥ body temperature ≥ 39 °C. Indirect calorimetry can be influenced by factors such as irritability, recent procedures, and potential issues like pain, which should be minimized as much as possible.

#### Data collection

The collected data included age, sex, weight (measured in kilograms), height (in centimeters), body mass index (BMI), primary reason for admission to the ICU, Acute Physiology and Chronic Health Evaluation (APACHE) II score, SOFA score, Nutrition Risk in the Critically Ill (NUTRIC) score, time from ICU admission to REE measurement, length of ICU stay, duration of mechanical ventilation, and the prognosis of individuals involved in the study. Simultaneously, the following parameters were collected: (1) Vital signs including body temperature (T), heart rate (HR), mean arterial pressure (MAP), diastolic blood pressure (DBP), and systolic blood pressure (SBP); and (2) REE parameters including REE, REE index (REEI), which represents the REE value relative to body weight, respiratory quotient (RQ), VO_2_, and VCO_2_.

#### Research methodology

This investigation employed a prospective, self-controlled research design. The study participants were individuals in a state of critical illness who required mechanical ventilation but maintained a lucid state of awareness. The study was conducted during the 72-h period following ICU admission, after clinical stabilization had been achieved. Remifentanil was used as an analgesic with the same analgesic target. Midazolam was used as the sedative agent. The depth of sedation was quantified using the Richmond Agitation-Sedation Scale (RASS). The REE of the study participants at different sedation levels on the same day was measured using the CARESCAPE R860 ventilator. Participants and their guardians were informed about the investigation and signed the informed consent document. The Ethics Committee of Jiangbei Hospital approved this study (number: 2023024).

#### Methods of sedation and analgesia administration

For all study participants, midazolam (Jiangsu Enhua Pharmaceutical Co., Ltd., National Drug Approval Number H20143222, specification 2 mL: 10 mg) was used to initiate and maintain sedation. The initial injection dose was 0.04 mg/kg, which was subsequently maintained at 0.04 mg/kg.h. The dose was adjusted by 20% every hour to attain the desired sedative level. The depth of sedation was assessed utilizing the RASS scale: mild sedation (RASS: 0 to −1), moderate sedation (RASS: −2 to −3), and severe sedation (RASS: −4 to −5). The sedation level was evaluated by the data collection personnel of the project and bedside nurses every 1 h. After reaching the sedation target, REE was measured using IC.

For all critically ill patients receiving mechanical ventilation, remifentanil (Jiangsu Enhua Pharmaceutical Co., Ltd., National Drug Approval Number H20143315, specification 1 mL: 2 mg) was used for analgesia treatment. The initial dose of remifentanil was 0.4ug/kg h, and the dose was increased or decreased by 50% every 1 h to reach the analgesia target.

#### Stress score criteria and analgesic target

The RASS scoring criteria are as follows (7): +4 points: The patient has violent impulses and exhibits strong aggressive behavior; +3 points: The patient is restless and uncooperative during the treatment process, such as attempting to remove the breathing tube or infusion tube by themselves; +2 points: The patient is severely restless, making ventilator use difficult during treatment; +1 point: The patient shows mild anxiety with minor body movements but can cooperate with treatment; 0 points: The patient is relatively calm and conscious; −1 point: The patient is not fully conscious during this period, showing a coma-like state, but can maintain consciousness for more than 10 s; −2 points: The patient cannot remain conscious for more than 10 s; −3 points: The patient responds to sounds; −4 points: The patient exhibits no reaction to auditory stimuli but responds to tactile stimuli; and −5 points: The patient exhibits no reaction to either auditory or tactile stimuli.

The Critical Care Pain Observation Tool is a scoring instrument for evaluating pain (8). Throughout the study, each participant was assigned an identical pain relief target: A CPOT score of < 3 points.

#### Assessment of REE

REE measurement was conducted using a ventilator. At the start of a specific test, the ventilator (CARESCAPE R860) was inserted into the slot, and its sampling tube and sensor were connected to each other and aligned with the patient’s breathing tube. The angle was slightly tilted. Then, the system was preheated for half an hour. Ventilator settings were kept unchanged for 90 min prior to the start of IC. The standard operating procedure for the test was as follows: The test lasted 30 min, but the recorded data did not include the first 5 min. If the coefficient of variation for VO_2_ and VCO_2_ remained below 10%, a REE value was considered valid.

### Statistical analysis

The collected data were analyzed using SPSS 26.00. Measurement data were assessed for normality. Normally distributed data were presented as mean ± SD. A *t*-test was performed to compare data across various categories. For data that did not follow a normal distribution, the median served as the representative measure, and the Mann–Whitney U test was used for group comparisons. The *χ*^2^ test was used for frequency analysis. Multiple linear regression was performed to evaluate the effects of different variables on REE. A *p*-value of less than 0.05 was considered statistically significant.

### Outcomes

#### General information

From September 2023 to September 2024, 74 individuals who were critically ill and required mechanical ventilation while remaining fully consciousness were included in the study. Among them, 45 were men (60.81%) and 29 were women (39.19%); their ages ranged from 25 to 83 years, with a median value of 68 years (IQR 58–79). The time of IC measurement (from the time of admission to the department) was 41 ± 7.6 h. The average amount of time spent on mechanical ventilation was 6 days (with a range of 4–10 days); the median length of ICU stay was 8 days (IQR 4–13). Among the 74 patients, 11 died in the ICU (14.86%). General patient characteristics are presented in Table 1.

**Table 1:** General patient characteristics.

Indicators	Data
Number of patients	74
Male, *n* (%)	45 (60.81%)
Female, *n* (%)	29 (39.19%)
Age [years, M (Q_L_, Q_U_)]	68 (52, 79)
Age, *n* (%)	
<45	9 (12.16%)
45–64	23 (31.08%)
>64	42 (56.76%)
Height [cm, M (Q_L_, Q_U_)]	167 (162, 174)
Weight [kg, M (Q_L_, Q_U_)]	64 (52, 71)
BMI [kg/cm^2^, M (Q_L_, Q_U_)]	21 (17.0, 24.6)
BMI, *n* (%)	
<18.5 kg/m^2^	12 (16.12%)
18.5–25.0 kg/m^2^	53 (71.62%)
>25.0 kg/m^2^	9 (12.16%)
APACHE II score at admission [points, M (Q_L_, Q_U_)]	19 (14, 26)
SOFA score at admission [points, M (Q_L_, Q_U_)]	7 (4, 12)
Diagnosis at ICU admission, *n* (%)	
Acute respiratory failure	31 (41.89%)
Acute heart failure	19 (25.68%)
After surgery	24 (32.43%)
NUTRIC score at ICU admission [points, M (QL, QU)]	6 (4, 9)
Low risk (0–4), *n* (%)	4 (5.56%)
High risk (5–9), *n* (%)	68 (94.44%)
Measurement time (from admission, h), mean (±SD)	41 (7.6)
ICU stay time [days, M (QL, QU)]	8 (4–13)
Mechanical ventilation time [days, M (QL, QU)]	6 (4–10)
ICU mortality rate, *n* (%)	11 (14.86%)

BMI, Body Mass Index; APACHE II, Acute Physiology and Chronic Health Evaluation II; SOFA, Sequential Organ Failure Assessment; NUTRIC, Nutrition Risk in the Critically Ill Score; ICU, Intensive Care Unit.

#### Comparison of REE indicators at different sedation levels

As sedation deepened, VCO_2_, VO_2_, REE/kg, and REE gradually decreased. VCO2 differed significantly among the sedation levels: Mild sedation (165.3 ± 13.8 mL/min), moderate sedation (150.7 ± 16.7 mL/min), and severe sedation (138.9 ± 12.1 mL/min) (*p* < 0.05). VO2 also showed significant differences: Mild sedation (138.9 ± 12.1 mL/min), moderate sedation (193.2 ± 21.5 mL/min), and severe sedation (178.1 ± 15.4 mL/min) (*p* < 0.05). There was a statistically significant difference in REE across the sedation levels: Mild sedation (1,347 ± 129 kcal/d), moderate sedation (1,236 ± 137 kcal/d), and severe sedation (1,347 ± 137 kcal/d) (*p* < 0.05). No statistically significant differences were observed in the RQ across the three groups (*p* > 0.05). Detailed data are presented in Table 2.

**Table 2:** Association between different levels of sedation and REE-related variables.

Sedation level	RQ	VCO_2_ (ml/min)	VO_2_ (ml/min)	REEI (kcal/kg.d)	REE (kcal/d)
Mild sedation	0.79 ± 0.04	165.3 ± 13.8	209.3 ± 17.2	22.1 ± 2.0	1,347 ± 129
Moderate sedation	0.78 ± 0.05	150.7 ± 16.7^a^	193.2 ± 21.5^a^	20.2 ± 2.1^a^	1,236 ± 137^a^
Severe sedation	0.78 ± 0.04	138.9 ± 12.1^ab^	178.1 ± 15.4^ab^	18.7 ± 1.7^ab^	1,140 ± 108^ab^

RQ, Respiratory Excess Volume; VCO_2_, Carbon Dioxide Production; VO_2_, Oxygen Consumption; REE/kg, Resting Energy Expenditure/Body Weight; REE, Resting Energy Expenditure. Compared to mild sedation, ^a^*p* < 0.05; Compared to moderate sedation, ^b^*p* < 0.05.

#### Distribution of participants across REEI ranges at various levels of sedation

Among the 74 patients, when REE was below 20 kcal/kg/d, mild, moderate, and severe sedation accounted for 54.05, 70.27, and 83.78% of patients, respectively. For REE levels between 20 and 25 kcal/kg/d, mild, moderate, and severe sedation accounted for 35.14, 24.32, and 14.86% of patients, respectively. When REE exceeded 25 kcal/kg/d, mild, moderate, and severe sedation accounted for 10.81, 5.41, and 1.35% of patients, respectively. Refer Table 3 for detailed information.

**Table 3:** Distribution of participants across REE/kg.d ranges at different levels of sedation.

REE/kg.d	<20.0 kcal/kg.d	20.0–25.0 kcal/kg.d	>25.0 kcal/kg.d
Mild sedation, *n* (%)	40 (54.05%)	26 (35.14%)	8 (10.81%)
Moderate sedation *n* (%)	52 (70.27%)	18 (24.32%)	4 (5.41%)
Severe sedation, *n* (%)	62 (83.78%)	11 (14.86%)	1 (1.35%)

#### Relationship between various levels of sedation and T, HR, MAP, sedation dosage, and analgesic dosage

There were no significant differences between various levels of sedation and T, HR, MAP, or the dosage of analgesics. However, there was a statistically significant association between various levels of sedation and the dosage of midazolam (*p* < 0.05), as displayed in Table 4.

**Table 4:** Differences in T, HR, MAP, sedation dosage, and analgesic dosage across sedation levels.

Degree of sedation	T (°C)	HR (per/min)	MAP (mmHg)	Midazolam (mg/kg.h)	Remifentanil (μg/kg.h)
Mild sedation	37.1 ± 0.5	84 ± 11	81 ± 13	0.04 ± 0.02	0.4 μg ± 0.02/kg.h
Moderate sedation	36.9 ± 0.6	81 ± 14	79 ± 12	0.07 ± 0.03^a^	0.4 μg ± 0.01/kg.h
Severe sedation	37.0 ± 0.4	82 ± 10	80 ± 14	0.15 ± 0.02^ab^	0.4ug ± 0.01/kg.h

Compared to mild sedation, ^a^*p* < 0.05; compared to moderate sedation, ^b^*p* < 0.05.

#### Logistic regression analysis of factors influencing REE

Multiple linear regression analysis was performed with REE as the dependent variable. We examined age, sex, BMI, sedation, APACHE II score, SOFA score, and NUTRIC score as potential influencing factors. Age, sex, BMI, and sedation were identified as independent factors influencing REE (*p* < 0.05), as illustrated in Table 5.

**Table 5:** Multiple linear regression analysis of factors influencing REE.

Factors	Β value	SE value	Wald *χ*^2^ value	*p*	OR	95% CI	VIP	Tol
Age	−0.195	0.162	7.362	0.018	0.672	0.264, 0.832	1.764	0.566
Male	3.355	0.533	6.534	0.035	2.151	1.623, 3.021	1.663	0.601
BMI	1.151	0.182	6.039	0.027	2.463	1.382, 3.419	2.538	0.387
Sedation	−0.214	0.224	9.151	0.011	0.427	0.312, 0.805	2.573	0.389
APACHE II score	0.157	0.101	0.532	0.214	1.182	1.029, 2.843	2.015	0.496
SOFA score	2.301	0.231	0.142	0.091	0.941	0.271, 1.519	1.240	0.806
NUTRIC score	0.176	0.132	0.985	0.753	1.062	1.002, 1.852	2.959	0.338

BMI, Body Mass Index; APACHE II, Acute Physiology and Chronic Health Evaluation II; SOFA, Sequential Organ Failure Assessment; NUTRIC, Nutrition Risk in the Critically Ill Score.

## Discussion

Nutrition is of vital importance for critically ill patients, and assessing energy requirements is the cornerstone of nutritional support therapy (9). Insufficient or excessive feeding can have negative clinical effects, including increased rates of infection and mortality (10). Conversely, individualized, targeted nutritional therapy is crucial for accelerating recovery and reducing complications in critically ill patients (11). However, determining the optimal energy requirement has become a major clinical issue. Currently, various predictive energy formulas are used to calculate energy requirements to guide nutritional implementation; however, the values calculated using these formulas are not as accurate as IC measurements. The reason for this discrepancy is that estimates based on population statistics do not accurately reflect an individual’s metabolic rate, and predicted values for critically ill patients often differ substantially from the actual values (12). IC-guided nutritional therapy can meet energy and protein needs, reduce mortality rates, and does not lead to an increased risk of adverse events (4). Consequently, the measurement of REE using IC is currently acknowledged as the gold standard for evaluating the energy needs of critically ill patients (13).

This study included critically ill patients with clear consciousness who required mechanical ventilation. This is because patients with clear consciousness can accurately quantify the depth of sedation using the RASS, while the RASS is of limited utility in patients with impaired consciousness (14). According to the 2023 clinical nutrition guidelines for ICUs, outlined by the European Society for Clinical Nutrition and Metabolism (ESPEN) (13), IC should be used to measure energy expenditure in critically ill patients receiving mechanical ventilation. As a result, the CARESCAPE R860 ventilator energy metabolism monitoring system was utilized to measure REE in critically ill patients requiring mechanical ventilation. Midazolam was selected as the sedative because of its rapid onset, quick metabolism, and minimal effects on hemodynamics (15).

This study showed that, among 74 patients with mild sedation, only 8 (10.81%) had REE/kg/d greater than 25 kcal/kg/d, 26 (35.14%) had REE/kg/d between 20 and 25 kcal/kg/d, and 40 (54.05%) had REE/kg/d less than 20 kcal/kg/d, with an average REE of 22.1 kcal/kg/d, which was lower than the energy requirement value recommended by the ESPEN (25 kcal/kg/d) (16). However, the results are consistent with the findings of Medeiros et al. (17). The lower REE observed in this study compared to the recommended value is likely related to the patients’ age, the early stage of illness, and the effects of sedation. A study by Pontzer showed that energy metabolism changes dynamically throughout life and, even with adjustments based on body composition, energy metabolism gradually decreases after the age of 60 (18). Magyar et al. found that younger age, male sex, and higher BMI are independent predictors of increased energy requirements (19). Preiser et al. reported that there is a reduction in enzyme activity and oxygen consumption during the initial phase of severe illness, accompanied by a decline in energy metabolism (20). In the initial phase of a critical medical condition, mitochondrial function is severely damaged, leading to a decrease in biosynthesis, increase in reactive oxygen species (ROS) production, and reduction in ATP synthesis by 50%. This results in reduced energy expenditure (21). The majority of patients in this study were older adults, all in the critical phase of severe illness and receiving sedation treatment, which may explain why the measured REE was lower than the recommended values. Multivariate logistic regression analysis showed that age, BMI, sex, and sedation were independent risk factors affecting REE, while the APACHE II score, SOFA score, and NUTRIC score had no correlation with REE.

Mechanical ventilation is a vital component in the therapeutic approach for critically ill patients (5). A survey conducted across 69 intensive care units in 14 countries, involving 2,088 patients on mechanical ventilation, showed (22) that 1,337 (64.0%) received benzodiazepines, 1,488 (70.9%) received propofol, 920 (44.1%) received dexmedetomidine, and 140 (6.7%) received ketamine for sedation. Sedation treatment can alleviate patients’ stress, restlessness, and anxiety, thereby facilitating the smooth implementation of monitoring and treatment in the ICU. This approach contributes to maintaining a safe and comfortable state for patients. For critically ill patients requiring mechanical ventilation, it is necessary to achieve the unification of sedation and mechanical ventilation, known as lung-protective sedation (6). Sedative agents are used to address human–machine incoordination, balance the risks and benefits of sedation, and reduce ventilator-related and spontaneous breathing-related lung injury, ultimately ensuring better lung protection (6). Therefore, different levels of sedation are required for various patients, conditions, and treatment responses in order to achieve specific sedation purposes.

**Figure 1:**
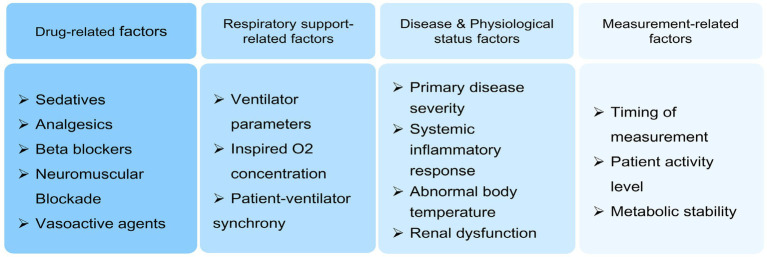
Factors influencing REE.

Critically ill patients, especially those who require mechanical ventilation, are influenced by multiple factors affecting REE, as shown in Figure 1. The impact of sedation treatment on patients’ REE has received increasing attention; however, findings remain inconsistent. Frankenfield and Koukiasa et al. suggested that sedation does not affect REE (23, 24), while McCall and Prange et al. found that sedation can reduce REE (25, 26). These discrepancies may be attributed to differences in study populations, research methods, sedation methods, sedation levels, and measurement timing. Using self-comparison before and after, this study examined the effects of varying levels of sedation on REE in critically ill patients requiring mechanical ventilation during the early stages of illness. The objectives were to reduce subject bias, ensure consistent sedation protocols, achieve target sedation levels, and maintain comparable measurement timing. Therefore, the study results are considered relatively reliable. The results showed that, as the sedative dose increased and the depth of sedation deepened, REE gradually decreased. Severe sedation resulted in a 15.38% decrease in REE (1,140 kcal/d) compared to mild sedation (1,347 kcal/d) and a 7.43% decrease compared to moderate sedation (1,236 kcal/d). This study suggests that, in critically ill patients requiring mechanical ventilation who remained conscious, REE gradually decreased with increasing depth of sedation during the early stage of illness.

The American Society for Parenteral and Enteral Nutrition (ASPEN) and the European Society for Clinical Nutrition and Metabolism (ESPEN) recommend indirect calorimetry as the gold standard for assessing REE in critically ill patients (13). However, the use of IC remains limited due to the substantial expenses associated with the required tools and consumables, as well as the lack of trained personnel to operate the devices. Moreover, not all patients are suitable candidates for the measurement of IC (27). A large prospective study found that only 0.8% of more than 8,000 documented cases underwent IC measurement (28). Therefore, many factors that may affect energy metabolism have been studied to correct the energy metabolism formula for nutritional support. This study confirmed that, in critically ill patients requiring sedation, when providing nutritional support treatment, the energy supply should be adjusted according to the depth of sedation to match the supply and demand.

The mechanism by which sedation affects REE is still unclear. Dorges’ study revealed that sedation can reduce excessive catecholamine release in the body, decrease oxygen demand, and enhance oxygen utilization (29). Oddo’s study found that sedation can reduce cerebral tissue oxygen metabolism in a dose-dependent manner (30). Therefore, it is hypothesized that sedation can reduce muscle activity, attenuate neuro-humoral responses to stress, decrease oxygen consumption, and lower tissue metabolism. Briesenick et al. (31) found that, in non-cardiac surgery patients, REE decreased by approximately one-quarter (953 kcal/m^2^.d vs. 680 kcal/m^2^.d, *p* < 0.001) after using analgesics, sedatives, and muscle relaxants. This study specifically investigated the effect of sedation on REE while maintaining analgesia, without using muscle relaxants. As a result, the REE of patients under severe sedation reduced by 15.37% compared to those under mild sedation, which is lower than the value reported in Briesenick’s study. This study attempted to control for potential confounding factors, including pain and irritability; however, there may still be other factors that could have impacted the results, which represents a limitation of this study. In addition, the time since ICU admission was not included as a covariate in the statistical analysis. Although all measurements were performed within a 72-h window following clinical stabilization, we acknowledge that the physiological progression of critical illness during this period may still influence metabolic parameters and potentially confound the effects of sedation. Future studies should include the time since admission as a covariate to better delineate these distinct contributions. Furthermore, due to the relatively small sample size, we were unable to perform multivariable regression analyses to adjust for potential confounders. Therefore, the observed association between sedation levels and REE should be interpreted as exploratory. Finally, the study did not systematically collect key clinical and metabolic variables, including the presence of infection, vasoactive agent use, acid–base status, renal function, and fluid balance. The absence of these data limits our ability to fully account for potential confounders and may affect the generalizability of our findings. Despite the presence of these influencing factors, the study indicates that the energy use of critically ill patients on mechanical ventilation is affected by sedation, with resting energy expenditure decreasing progressively as the level of sedation increases.

## Conclusion

In conclusion, under rigorously controlled and reproducible ventilatory conditions—including constant mode, PEEP, FiO₂, and tidal volume, with ventilatory parameters included as covariates in the statistical analysis to isolate their effect—sedation was found to significantly influence REE in critically ill patients receiving mechanical ventilation. REE decreased progressively with increasing sedation intensity, and this effect was independent of ventilator settings. Therefore, the impact of sedation depth on the metabolic rate should be taken into account when individualizing nutritional therapy for this patient population. Future large-scale, multicenter, prospective studies are warranted to further validate these findings and to explore potential differences among various sedative agents and distinct critical illness subgroups.

## Data Availability

The original contributions presented in the study are included in the article/supplementary material, further inquiries can be directed to the corresponding author/s.
